# Patients perspectives on drug shortages in six European hospital settings – a cross sectional study

**DOI:** 10.1186/s12913-021-06721-9

**Published:** 2021-07-12

**Authors:** Darija Kuruc Poje, Domagoj Kifer, Isabelle Huys, Joao Miranda, Helena Jenzer, Nenad Miljković, Torsten Hoppe-Tichy, Marcin Bochniarz, Roberto Frontini, David G Schwartz, Vesna Vujić-Aleksić, Lana Nežić, Eleni Rinaki, Leonidas Tzimis, Kim Green, Jelena Jovanić, Bojana Carić, Danijela Mandić, Katarina Vilić, Tomasz Bochenek, Vesna Bačić Vrca, Srećko Marušić

**Affiliations:** 1 Pharmacy Department, General hospital “dr. Tomislav Bardek”, Koprivnica, Croatia; 2 Department of Biophysics, Faculty of Pharmacy and Biochemistry, University of Zagreb, Zagreb, Croatia; 3 Department of Pharmaceutical and Pharmacological Sciences, KU Leuven, Leuven, Belgium; 4 Departamento de Tecnologias, Escola Superior de Tecnologia e Gestão, Instituto Politécnico de Portalegre, Portalegre, Portugal; 5 CERENA - Centro de Recursos Naturais e Ambiente, Instituto Superior Técnico, Universidade de Lisboa, Lisboa, Portugal; 6 Berner Fachhochschule Health Professions Ernährung und Diätetik, Zürich, Switzerland; 7 Pharmacy Department, Institute of Orthopaedic Surgery “Banjica”, Belgrade, Serbia; 8 Pharmacy Department, Heidelberg University Hospital, Heidelberg, Germany; 9 Specialist Hospital Brzozów, Podkarpackie Oncological Center, Brzozów, Poland; 10 European Association of Hospital Pharmacists (EAHP), Brussels, Belgium; 11 School of Business, Bar-Ilan University, Ramat Gan, Israel; 12 Faculty of Medicine, University of Banja Luka, Banja Luka, Bosnia and Herzegovina; 13 Hospital pharmacy, Chania General Hospital “Saint George”, Crete Chania, Greece; 14 Department of Drug Management, Faculty of Health Sciences, Jagiellonian University Medical College, Kraków, Poland; 15 Pharmacy Department, Clinical hospital Dubrava, Zagreb, Croatia; 16 Endocrinology Department, Clinical hospital Dubrava, Zagreb, Croatia

**Keywords:** Drug shortages, Patient safety, Hospital setting, Patients’ perspectives, Europe

## Abstract

**Background:**

It is known that drug shortages represent a major challenge for all stakeholders involved in the process, but there is little evidence regarding insights into patients′ awareness and perspectives. This study aimed to investigate the patients-perceived drug shortages experience and their view on outcomes in different European hospital settings. Furthermore, we wanted to explore information preferences on drug shortages.

**Methods:**

A retrospective, cross sectional, a mixed method study was conducted in six European hospital settings. One hospital (H) from each of this country agreed to participate: Bosnia and Herzegovina (H-BiH), Croatia (H-CR), Germany (H-GE), Greece (H-GR), Serbia (H-SE) and Poland (H-PO). Recruitment and data collection was conducted over 27 months from November 2017 until January 2020. Overall, we surveyed 607 patients which completed paper-based questionnaire. Questions related to: general information (demographic data), basic knowledge on drug shortages, drug shortages experienced during hospitalization and information preferences on drug shortage. Differences between hospital settings were analyzed using Chi-squared test or Fisher’s exact test. For more complex contingency tables, Monte Carlo simulations (N = 2000) were applied for Fisher’s test. Post-hoc hospital-wise analyses were performed using Fisher’s exact tests. False discovery rate was controlled using the Bonferroni method. Analyses were performed using R: a language and environment for statistical computing (v 3.6.3).

**Results:**

6 % of patients reported experiences with drug shortages while hospitalized which led to a deterioration of their health. The majority of affected patients were hospitalized at hematology and/or oncology wards in H-BiH, H-PO and H-GE. H-BiH had the highest number of affected patients (18.1 %, N = 19/105, *p* < 0.001) while the fewest patients were in H-SE (1 %, *N* = 1/100, *p* = 0.001). In addition, 82.5 %, (*N* = 501/607) of respondents wanted to be informed of alternative treatment options if there was a drug shortage without a generic substitute available. Majority of these patients (66.4 %, N = 386/501) prefer to be informed by a healthcare professional.

**Conclusions:**

Although drug shortages led to serious medical consequences, our findings show that most of the patients did not perceive shortages as a problem. One possible interpretation is that good hospital management practices by healthcare professionals helped to mitigate the perceived impact of shortages. Our study highlights the importance of a good communication especially between patients and healthcare professionals in whom our patients have the greatest trust.

**Supplementary Information:**

The online version contains supplementary material available at 10.1186/s12913-021-06721-9.

## Background

Medicine shortages represent a significant public health problem that deserves the joint attention of governments and industries [1–3]. An increasing number of studies over the past decade report a higher frequency of drug shortages that lead to a high burden of the long-term supplies of key medicines worldwide [1–10]. As the World Health Organization (WHO) has stated, on top of additional costs for health systems, shortages pose risks to the health of patients who fail to receive the medicines they need. This leads to an increased risk of medication errors, adverse drug events or even death [11]. Norepinephrine shortage is one of the best example of that, as is it led to higher in-hospital mortality in patients with septic shock despite the available alternative [12]. In order to reduce these effects, clinicians routinely operate in crisis mode [2]. For instance, the European Association of Hospital Pharmacists (EAHP) found that an increase in drug shortages from 2014 to 2019 had a serious impact on hospital pharmacists’ workload [4, 13–15]. EAHP further found that an overwhelming majority of hospital pharmacists (86 % − 2014; 92 % − 2018; 95 % − 2019) reported that they had current problems with drug shortages in terms of delivering the best care to patients and/or operating the hospital pharmacy. The most affected therapeutic areas were infectious diseases, oncology, emergency medicine, cardiovascular medicine and anesthesia. Similar findings have been reported in Australia, Canada and the USA [2, 3, 8, 9, 12, 16]. Furthermore, the shortage of one medicine has the potential to expand to other generic substitutes or alternatives, and severely limit patient care despite the best mitigation efforts of hospital pharmacists and other healthcare workers [16, 17].

Drug shortages represent a major challenge for all stakeholders involved in the process of providing medication (e.g., manufacturers, supply chains, healthcare providers, patients). They lead to significant healthcare burden. The cost and time related to the labor it takes for hospitals to manage drug shortages and maintain quality patient care is 216 million US dollars per year in additional labor costs for pharmacists, pharmacy technicians, physicians, nurses [2]. Consequences of drug shortages also include: increases in drug budget; lost revenue from cancelled infusions and procedures; increased numbers of fulltime pharmacy and technician employees; reallocation of pharmacy resources, which leads to lost productivity and impact in other areas; negative impact on patient care [18]. Among all the problems that stem from drug shortages, patient safety stands out as the most crucial and critical [1, 19–21]. Recent research has demonstrated that patients’ perspectives are also important [21]. Michaud et al. investigated experiences of 471 patients with rheumatic diseases in the United States during the COVID-19 pandemic, which led to a hydroxychloroquine shortage [22]. They found that many patients thought that use of immunosuppressive medications increased their risk/potential severity of COVID‐19 and that stopping such medications could reduce this risk [22]. The study found that as a result, some patients altered their medications without professional consultation and others because of a hydroxychloroquine shortage. This is a significant finding, as clinicians have to be aware when patients stop taking their medications without a recommendation from a health professional. This highlights the need for studies with insights into patients′ awareness and perspectives which can quantify the effect of drug shortages on patient outcomes including in hospital settings. Many studies have been conducted in Canada and the USA while in Europe researchers have rarely studied the patients’ perspective from both a clinical impact and patient opinion perspective [2, 4, 5, 9, 12, 14, 15, 22–30]. Therefore the aim of this study was gathering contemporary data on patients-perceived drug shortages experience and their view on outcomes in different European hospital settings taking into account a humanistic approach. In addition, we wanted to explore information preferences on drug shortages.

## Methods

### Study design

We conducted retrospective, cross sectional mixed method study in six hospital settings in six European countries. The questionnaire consisted of combined quantitative multiple choice and qualitative open-ended questions. Open-ended questions were included to better explore diverse patients’ knowledge on drug shortages and eventually find relationship between their perspectives and outcomes.

### Setting

From six hospital settings that agreed to participate (located in Bosnia and Herzegovina (H-BiH), Croatia (H-CR), Germany (H-GE), Greece (H-GR), Serbia (H-SE) and Poland (H-PO)) two are university hospitals (H-BiH and H-GE), two general hospitals (H-CR and H-GR) while two are specialist hospitals (H-SE and H-PO). Characteristics of hospitals are shown in Table 1.

**Table 1:** Characteristics of hospitals

Characteristics						
Country of hospital setting	Bosnia and Herzegovina (H-BiH)	Croatia (H-CR)	Germany (H-GE)	Greece (H-GR)	Serbia (H-SE)	Poland (H-PO)
Functionality	Tertiary care university hospital	Secondary care general university hospital	Tertiary care university hospital	Secondary care general university hospital	Tertiary care specialist hospital	Tertiary care specialist hospital
Type of care and specialization	Acute general	Acute general	Acute general	Acute general	Chronic orthopedic	Chronic oncology
Number of beds	1198	350	2000	650	550	480
Municipality covered by hospital	Whole Rebublic Srpska in Bosnia and Herzegovina	Municipality of Koprivnica-Križevci	City of Heidelberg	Municipality of Chania	Whole country of Republic of Serbia	Municipality of Brzozow, Podkarpackie voivodship
Number of inhabitants in the municipality covered by hospital	1 142 495	111 782	160 355	108 642	6 945 000	65 000
Ownership / type of health system	Public	Public	Public	Public	Public	Public

### Participants

Participants of COST CA15105^1^ action and EAHP members were invited to take part in this study via meetings, emails and personal contacts. A cover letter explaining the aim of this study together with a patient friendly leaflet and questionnaire was provided. To improve the response rate a principal investigator with the help from EAHP staff, sent reminders for the study via email, an online information platform (EAHP monitor) and social networks. We used non-probability convenience sampling method because it is the least expensive, the least time consuming and one of the most convenient method to use. Additionally, due to limited time that researchers have in their everyday practice and to gather as many different hospitals which are different in size, we decided to have minimal number of respondents per hospital (*N* = 100). Moreover, we expected similar answers from respondents. Recruitment and data collection was conducted over 27 months from November 2017 until January 2020. The extended data collection period was to allow for a wide range of naturally occurring medication shortages, and to enable a large and geographically distributed selection of hospitals to participate. The role of investigators who agreed to participate in the study was to gather approval from the Ethics committee from their hospital, translate questionnaire to their language following guidelines of the WHO^2^, to recruit patients with explanation of the purpose of the study and to enter the patients’ answers into computer base that was created specifically for this study in English language.

Patients completed the survey by paper. In most cases, the survey was administered at hospital discharge while the patient was awaiting his/her discharge letter. Inclusion criteria included at least one day of hospitalization (overnight, inpatient), and being of age 18 years or older.

### Questionnaire

As we did not find questionnaire in the literature that met our expectations, we designed questions based on our professional experiences in dealing with drug shortages. This also included gathering patients’ opinions on what they consider important when drug shortages occur. At first stage two authors of the study developed a questionnaire which was checked by two experts in drug shortages. Questionnaire was then modified and adapted to their suggestions which they checked again and approved. Subsequently, two other experts and non-experts in this field corroborated the questionnaire. After final approval, author of the study validated questionnaire on convenience sample of 50 patients (even distribution of both sexes). The validation of the study was done by answering (yes or no) to following questions: if the questions were clear and easy, if the questions covered all problem areas within understanding of proposed “patient friendly” definition on drug shortages, if questionnaire does not lack important questions regarding patients perspectives on drug shortages and if questions do not violate patients privacy. Final questionnaire consisted of sixteen main questions and eight sub-questions (altogether twenty four questions). Fourteen of these were single-selection multiple-choice questions, one was a multi-selection multiple choice question, three were dichotomous questions and six were open-ended. Questions related to general information (demographic data), basic knowledge on drug shortages, drug shortages experienced during hospitalization and information preferences on drug shortage. Furthermore, as authors and pilot tested patients agreed on unambiguous and easy to comprehend drug shortage definition and questions, authors concluded that the questionnaire is reliable. The full questionnaire is available in the supplementary material.

### Definition

For the purpose of this study, we used the following “patient friendly” definition: “A drug shortage is insufficient supply (e.g., from a hospital pharmacy) of a drug that you (a patient) are currently taking without a generic substitute. A generic substitute is a medicine with the same active substance as the drug you (a patient) are taking but produced by another manufacturer.”

### Ethics committee and informed consent

The General hospital “dr. Tomislav Bardek” Koprivnica”, Croatia, ethics committee (Institutional Review Board) as well as other hospitals (H-BiH, H-GE, H-GR, H-SE and H-PO) that participated in the study approved all aspects of the study and questionnaire. All methods were carried out in accordance with relevant guidelines and regulations. Survey administration was preceded by informed consent. All participant information obtained during the study was kept confidential.

### Statistical analysis

All data are presented in absolute and relative frequencies. Differences are between hospital settings and not between countries. The frequencies of obtained answers were compared with each other between different hospitals. These differences were analyzed using chi-squared test, unless required assumptions were not met (i.e. small sample size) in which case Fisher’s exact test was used. For more complex contingency tables, Monte Carlo simulations (*N* = 2000) were applied for Fisher’s test. Post-hoc hospital-wise analyses were performed using Fisher’s exact tests and false discovery rate was controlled using the Bonferroni method. For age, trends were analyzed using the Cochran-Armitage trend test. Analyses were performed using *R: a language and environment for statistical computing (v 3.6.3)* [31]. We gave patients ' opinion as a textual answer. As this approach is extremely difficult to analyze and objectively present as size or number and compare attitudes between people, Word cloud was used as the best available option. This graphically shows exactly what patients were thinking and talking about when asked about drug shortages.

## Results

### Patients′ characteristics

A total of 607 questionnaires were gathered from six hospitals: H-BiH (*N* = 105), H-CR (*N *= 113), H-GE (*N *= 69), H-GR (*N* = 116), H-SE (*N* = 100) and H-PO (*N* = 104). From answered questionnaires, 204 and 221 patients did not answer to questions referring to their own definition on drug shortages and preference on information on drug shortages (questions 7 and 16 b)). We excluded hospital from France because of small number of recruited patients (*N* = 18). There was a balanced distribution of female (52 %) and male (48 %) patients′ answers. Most patients (44 %, *N* = 264/607) were between 46 and 65 years (Table 2). Occupation data is provided in the supplementary material. More than half of patients (51 %, *N* = 310/607) were hospitalized in a surgical ward, almost half (45 %, *N* = 274/607) in a non-surgical ward and 4 % (*N* = 23/607) could not identify the ward in which they were hospitalized. Almost one tenth (9 %, *N* = 53/607) of patients were transferred from another hospital to support continued medical treatment. Detailed tables are available in the supplementary material.

**Table 2:** Sex and age - comparisons are made between hospitals

Country, where hospital is located	Female N (%)	Male N (%)	18–25 years N (%)	26–45 years N (%)	46–65 years N (%)	66–80 years N (%)	81–95 years N (%)	96 and older N (%)
Greek hospital (H-GR)	77 (66)	39 (34)	10 (9)	41 (35)	29 (25)	26 (22)	10 (9)	0 (0)
Croatian hospital (H-CR)	44 (39)	69 (61)	0 (0)	13 (12)	58 (51)	34 (30 )	8 (7)	0 (0)
Bosnian and Herzegovinian hospital (H-BiH)	50 (48)	55 (52)	6 (6)	27 (26)	41 (39)	27 (26)	4 (4)	0 (0)
Polish hospital (H-PO)	55 (53)	49 (47)	3 (3)	21 (20)	53 (51)	26 (25)	1 (1 )	0 (0)
Serbian hospital (H-SE)	65 (65)	35 (35)	10 (10)	14 (14)	52 (52)	23 (23)	1 (1)	0 (0)
German hospital (H-GE)	26 (38)	43 (62)	2 (3)	5 (7)	31 (45)	22 (32)	9 (13)	0 (0)
Total N (%)	317 (52)	290 (48)	31 (5)	121 (20)	264 (44)	158 (26)	33 (5)	0 (0)

### Patient-perceived drug shortages experience and view on outcomes

**Fig. 1:**
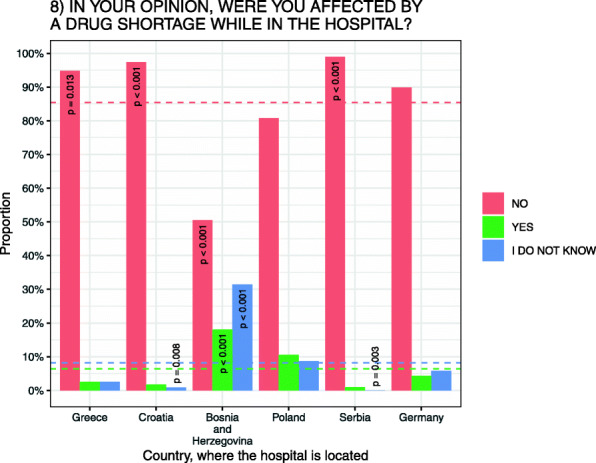
Patient-perceived drug shortages while hospitalized

Patents reported if they believed they had been affected by a drug shortage while hospitalized. Of the 607 patients that participated in the study, 6.4 % (*N* = 39/607) believed they were affected by a drug shortage while in hospital, 8.2 % (*N* = 50/607) did not know and 85.4 % (*N* = 518/607) believed they were not affected (Fig. 1). In the 39 cases of perceived drug shortage, most (51 %; *N* = 20/39) were hospitalized in hematology and/or oncology wards in H-BiH, H-PO and H-GE.

The hospital with highest level of patient-perceived drug shortage was H-BiH with 18.1 % (N = 19/105), with most patients hospitalized in a hematology and oncology ward and in a coronary and cardiology ward (*p* < 0.001). The lowest level of patient-perceived drug shortages was in H-SE with 1 % (*N* = 1/100) hospitalized in a spinal surgery ward (*p* = 0.001).

The vast majority (95 %, *N* = 37/39) of hospitalized patients that perceived they were affected by a drug shortage were informed about it by a healthcare professional.

In most cases of perceived shortage, if there was no available substitution for the drug, treatment was postponed (59 %, *N* = 23/39). The highest level of postponements occurred in hematology and/or oncology ward in H-BiH (26.1 %, *N* = 6/23) and H-PO (30.4 %, *N* = 7/23). Lack of a substitute drug resulted in treatment cancellation in three cases (7.7 %, *N* = 3/39). Patients were hospitalized in the hematology and/or oncology ward in H-BiH and H-PO, while in H-GE patient was hospitalized in the orthopedic ward.

**Fig. 2:**
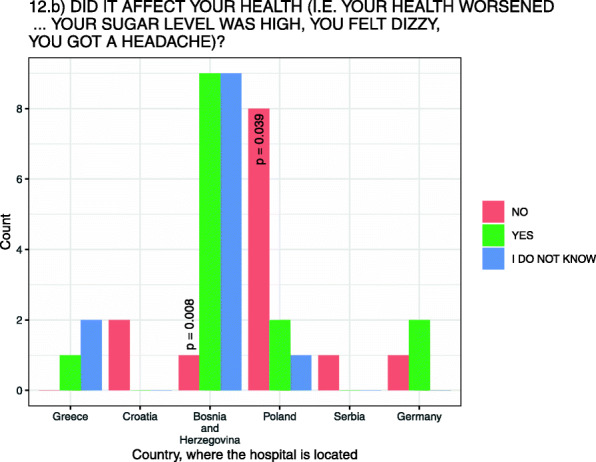
Patient-perceived drug shortage impact on their health

Patients who believed they had been affected by a drug shortage were asked if they believed the drug shortage had negatively affected their health. Of the 35.9 % (*N* = 14/39) of patients who answered that drug shortages had a negative effect on his or her health (e.g., health worsened) (Fig. 2), two thirds of patients (64.3 %, *N* = 9/14) were from H-BiH with most (*N* = 5) patients hospitalized in hematology and oncology ward.

**Fig. 3:**
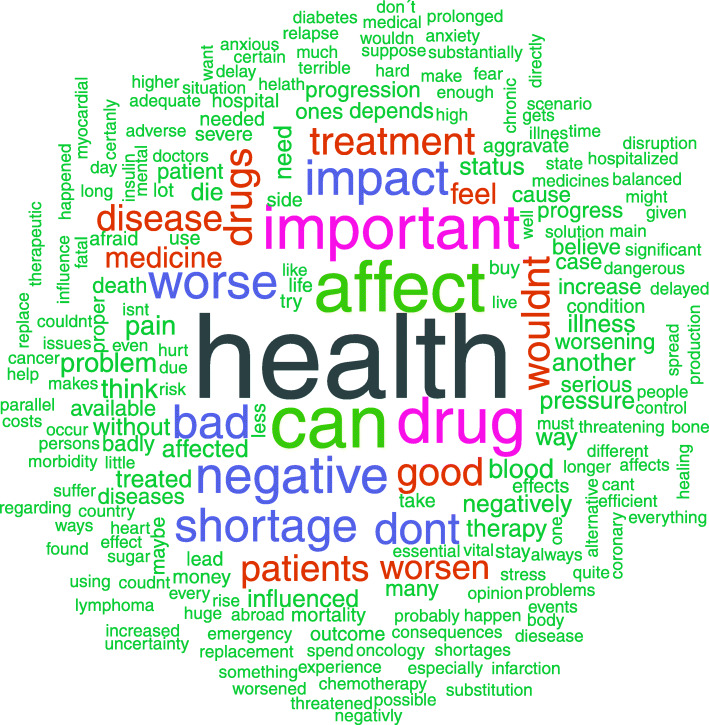
Opinions of patients on impact of drug shortages on their health

An open question allowing patients to express their opinion on how a drug shortage had or might affect their health, was answered by all 607 respondents. The text of these answers was translated into English using Google translate, validated by the co-investigators and aggregated to produce an illustrative word cloud (Fig. 3) that emphasizes the most common terms used.

### Willingness to be informed

Overall, most patients (82.5 %, *N* = 501/607) from all six hospital settings wanted to be informed of alternative treatment options if there was a drug shortage without a generic substitute available. Majority of patients (66.4 %, *N* = 386/501) prefer to be informed by a healthcare professional. Country-specific affirmative response rates from six hospital settings were: Serbia 73 % (*N* = 73/100); Croatia 76.1 % (*N* = 86/113); Poland 78.8 % (*N* = 82/104); Germany 87 % (*N* = 60/69), Bosnia and Herzegovina (90.5 %, *N* = 95/105) and Greece 90.5 % (*N* = 105/116).

**Fig. 4:**
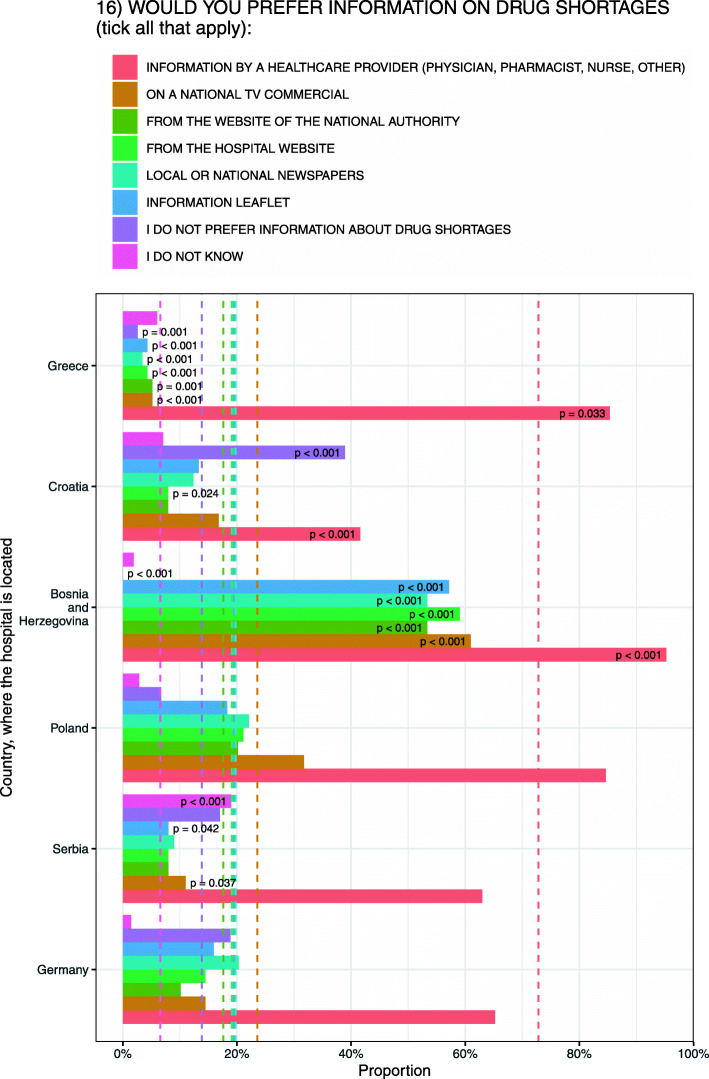
Patients’ preferences on drug shortage information channels

Patients differ in their preferences for receiving information on drug shortages. Figure 4 summarizes patients’ preferences on drug shortage information.

## Discussion

### Patient-perceived drug shortages experience and view on outcomes

Aim of this study was to investigate the patients-perceived drug shortages experience and their view on outcomes in different European hospital settings. Our results indicated that, despite an increasing number of studies reporting higher frequency of drug shortages over the past several years [1–10, 13–15], only 6 % of patients thought they were affected by drug shortages while hospitalized. This provides new insight into the relationship between hospital pharmacists and other healthcare professionals, who appear to be taking measures to successfully mitigate shortage impact on patient care. The indicated finding is important as healthcare and hospital policies are often inadequate (e.g. current shortage lists are often not providing the real-time shortage information or due to the legal complexities of drug borrowing/distribution, borrowing is restricted to urgent situations and never resolves the shortage) [32]. Consequently, institutions often response in isolation, uncoordinated with each other and management may differ between them. Moreover, hospitals often use different approaches to certain shortages and can be unaware of shortages their neighbors are facing [2].

One of the few studies that reported patient perceptions in Europe was the EAHP 2019 survey [15]. It showed higher frequency of drug shortages, but the question also involved patients′ family members who were not hospitalized at the time of the study. Moreover, it included generic substitutes or original medicine that the patient or his/her family member was taking but in lower dose (e.g., “There were patients that were advised to take more pills from a lower dose.”) [15]. Our study specifically addressed just patients at the day of discharge from hospital and shortages of drugs that did not have generic substitutes.

We note that according to the 2018 EAHP survey [14] Bosnia and Herzegovina has been identified as the country most affected by drug shortages, in terms of both mean duration and frequency. This provides support to our findings of high levels of patient-reported shortages occurring in that country.

Despite efforts by healthcare professionals to mitigate drug shortages, they often cannot be mitigated. Such was the case in more than half (51 %) of the affected patients hospitalized at hematology and/or oncology wards. Furthermore, our results should be taken into account when considering that antimicrobial, oncology, emergency and critical care drugs are increasingly unavailable due to pricing or withdrawal from the market. In addition, when they are withdrawn from the market for reasons other than safety or efficacy, there are limited mechanisms to ensure they remain available to patients that need them [3, 7, 12, 17, 33, 34]. This is especially true for those hospitalized in hematology and oncology wards where therapy is time dependent and any delay or cancellation can lead to serious long-term consequences [1, 25, 33, 35, 36]. Healthcare providers appear to be well aware of these problems [3, 4, 8–10, 12–15, 25, 28–30] as reflected in their role of providing shortage information to affected patients (95 %).

The study demonstrates a correlation between postponed treatment and perceived drug shortages in almost two thirds (59 %) of cases when there was no substitution for the patients’ drug while hospitalized. This confirms similar findings from other studies that reported shortages from the healthcare providers’ perspective [14, 23, 25, 28]. In addition, our findings are in line with a recent systematic review of 40 studies by Phuong et al. that explored predominantly negative impacts of medication shortages on the economic, clinical, and/or humanistic outcomes of patients [37]. However, this review included just a single study of the 40 that examined patients’ perspectives on medication shortages, and that study had a single-medication focus (antiepileptic clobazam) while our study considered all affected medicines in shortage.

Notwithstanding the fact that most supply disruptions and drug shortages occur in areas where a good alternative therapy is available, with minimum health implications for patients [19], our results demonstrated drug shortages have clinical significance from the patients point of view as they confirmed that their health worsened due to postponed or cancelled treatment. In addition, this correlates with other respondents who did not experience drug shortages but gave the opinion that shortages could have negative affect on their health.

### Willingness to be informed

This study shows the importance of patients being made aware that there are alternative treatment options when no generic substitute is available. Although, withholding information on drug shortages is done primarily to avoid causing additional patient anxiety, particularly if only minor effects are anticipated [38], informing patients of the alternative treatment process may lead to better outcomes. One example is a research by van Langenberg et al. that showed switching to alternative treatment for ulcerative colitis could have significant impact on patient care with more adverse events (abdominal pain, hepatotoxicity, nausea, hypersensitivity reaction). Patients were actively participating in the study by reporting these adverse events, having been made aware that they were receiving an alternative treatment. All of them had prompt resolution of symptoms upon cessation of alternative therapy [39]. By prioritizing these patients for original treatment when available, possibility of therapy discontinuation and therefore health deterioration is reduced to a minimum.

We suggest that the reason patients from H-BiH wanted to be informed of drug shortages through all possible information channels is because of the high levels of patient awareness of drug shortages. In contrast to them, patients from H-CR had the least desire to be informed due to less number of affected patients by drug shortages. Lower levels of awareness and not understanding the problem, combined with impatience to be discharged from the hospital as soon as possible, may have resulted in a bias leading to lack of response to the final question of the questionnaire. Moreover, despite the fact that patients from H-SE was the hospital with fewest affected patients, they did not know if they wanted to be informed about drug shortages while hospitalized. This could be due to the immediate impact to awareness caused by participating in this survey, but not being able to decide if this is important or not as had not experienced it.

Our study highlights the importance of a good communication especially between patients and healthcare professionals in whom our patients have the greatest trust. To support this, regulatory authorities should establish carefully planned communication strategies using various tools. These could be the ones preferred by patients in our study such as local or national press releases, television, information leaflets, and websites of national authority.

### Limitations and recommendations for the future

Our primary goal was understanding the perspective of hospitalized patients and this study is not representative of a general population or a general patient population. Due to convenience sampling, after achieving required or almost required minimal number of participants, investigators stopped collecting further data. This explains limitation of our sample size and further studies should survey larger patient populations. While each ethics committee approval was received at each hospital where this survey was administered, a number of hospitals declined approval fearing that interviewed patients would become distressed at the prospect of there having been a drug shortage and may lose trust in the hospital system. Future studies should attempt to address this concern and thus broaden participation. While previous research has focused primarily on hospital pharmacists, our results revealing patients’ insights can help create a more robust assessment of and response to drug shortages. We focused solely on the perspectives of hospitalized patients and further studies should include/compare with the perspectives of patients in the community.

## Conclusions

It is important to better understand relationships that might exist between patients′ opinions and their acts (e.g., stop taking medicine). Patients’ actions are often influenced by their perceptions of the medical environment and the care that they receive.

Healthcare professionals, especially hospital pharmacists, are on the frontline of drug shortages where they must alleviate the impact on patient care. The fact that only 6 % of patients reported feeling affected by drug shortages without generic substitution is a strong indication that healthcare professionals are successfully mitigating shortages. A further optimistic finding is that 95 % of patients experiencing shortages felt that shortage information was properly delivered by healthcare professionals. Unfortunately, most of the patients affected by drug shortages, hospitalized at hematology and/or oncology wards in H-BiH, H-PO and H-GE, and did not have an alternative solution. They reported that their health worsened. H-BiH was the most affected. This finding highlights the complexity of the problem, the lack of systemic awareness of drug shortages, and lack of adequate attention from national regulatory bodies in European countries. Establishing systematic, timely and transparent reporting is the foundation of a patient-centered approach to drug shortages. Information provided through various communication channels should provide patients with practical and understandable ways that shortages are being mitigated. Drug shortages will continue to impact patient care however, the approaches we have uncovered and advocated in this study could help mitigate the issue by actively involving patients as a part of a systemic solution.

## Supplementary Information

**Additional file 1.**

**Additional file 2.**

## Data Availability

The datasets generated during and/or analysed during the current study are available from the corresponding author on reasonable request.

## Abbreviations

COVID-19: Coronavirus disease 2019
EAHP: European Association of Hospital Pharmacists
H: Hospital
H-BiH: Hospital that participated in the study from Bosnia and Herzegovina (University Clinical Centre of Republic of Srpska, Banja Luka)
H-CR: Hospital that participated in the study from Croatia (General hospital “dr. Tomislav Bardek”, Koprivnica)
H-GE: Hospital that participated in the study from Germany (Heidelberg University Hospital, Heidelberg)
H-GR: Hospital that participated in the study from Greece (Chania General Hospital “Saint George”, Chania, Crete)
H-SE: Hospital that participated in the study from Serbia (Institute of Orthopaedic Surgery “Banjica”, Belgrade)
H-PO: Hospital that participated in the study from Poland (Specialist Hospital Brzozów, Podkarpackie Oncological Center, Brzozów)
USA: United States of America
WHO: World Health Organization

## Footnotes

1 https://www.cost.eu/actions/CA15105/#tabs/Name:overview.

2 Process of translation and adaptation of instruments. link: https://www.who.int/substance_abuse/research_tools/translation/en/
