# Supplementary material for: Patients perspectives on drug shortages in six European hospital settings – a cross sectional study
Source: BMC Health Serv Res. 2021 Jul 12;21:689. doi: 10.1186/s12913-021-06721-9 (PMC8274960; doi:10.1186/s12913-021-06721-9)
Supplement: Supplementary file 1 — Additional file 1. [file 12913_2021_6721_MOESM1_ESM.docx]

**Patients Perspectives on Drug Shortages in six European hospital settings**

**Darija Kuruc Poje^1*^, Domagoj Kifer^2^, Isabelle Huys^3^, Joao Miranda^4,5^, Helena Jenzer^6^, Nenad Miljković^7^, Torsten Hoppe-Tichy^8^, Marcin Bochniarz^9^, Roberto Frontini^10^, David G Schwartz^11^, Vesna Vujić-Aleksić^12,13^, Lana Nežić^13^, Eleni Rinaki^14^, Leonidas Tzimis^14^, Kim Green^8^, Jelena Jovanić^15,16^, Bojana Carić^15, 17^, Danijela Mandić^15,18^, Katarina Vilić^1^, Tomasz Bochenek^19^, Vesna Bačić Vrca^20^, Srećko Marušić^21^**

1. Pharmacy Department, General hospital “dr. Tomislav Bardek”, Koprivnica, Croatia
2. Department of Biophysics, Faculty of Pharmacy and Biochemistry, University of Zagreb, Zagreb, Croatia
3. Department of Pharmaceutical and Pharmacological Sciences, KU Leuven, Leuven, Belgium
4. Departamento de Tecnologias, Escola Superior de Tecnologia e Gestão, Instituto Politécnico de Portalegre, Portalegre, Portugal
5. CERENA - Centro de Recursos Naturais e Ambiente, Instituto Superior Técnico, Universidade de Lisboa, Lisboa, Portugal
6. Berner Fachhochschule Health Professions Ernährung und Diätetik, Zürich, Switzerland
7. Pharmacy Department, Institute of Orthopaedic Surgery “Banjica”, Belgrade, Serbia
8. Pharmacy Department, Heidelberg University Hospital, Heidelberg, Germany
9. Specialist Hospital Brzozów, Podkarpackie Oncological Center, Brzozów, Poland
10. European Association of Hospital Pharmacists (EAHP), Brussels, Belgium
11. School of Business, Bar-Ilan University, Ramat Gan, Israel
12. Republic of Srpska Agency for Certification, Accreditation and Quality Improvement in Health Care, Banja Luka, Bosnia and Herzegovina,
13. Department of Pharmacology, Toxicology and Clinical Pharmacology, Faculty of Medicine, University of Banja Luka, Banja Luka, Bosnia and Herzegovina
14. Hospital pharmacy, Chania General Hospital “Saint George”, Chania, Crete, Greece
15. Faculty of Medicine, University of Banja Luka, Banja Luka, Bosnia and Herzegovina
16. Department of Cardiology, University Clinical Centre of Republic of Srpska, Banja Luka, Bosnia and Herzegovina
17. Department of Endocrinology, University Clinical Centre of Republic of Srpska, Banja Luka, Bosnia and Herzegovina
18. Department of Hematology, University Clinical Centre of Republic of Srpska, Banja Luka, Bosnia and Herzegovina
19. Department of Drug Management, Faculty of Health Sciences, Jagiellonian University Medical College, Kraków, Poland
20. Pharmacy Department, Clinical hospital Dubrava, Zagreb, Croatia
21. Endocrinology Department, Clinical hospital Dubrava, Zagreb, Croatia

*** Correspondence:**Darija Kuruc Poje, darija_kuruc@yahoo.com, ORCID ID: 0000-0002-7893-786X

**PATIENT SURVEY ABOUT DRUG SHORTAGES**

**Drug shortages are a global problem and this can result in a significant impact for the patient. Although the importance of the problem, no objective data are available on the patients impact. This survey aims to quantify the impact of drug shortages at patient level in hospital environment in __________________________ region . Filling in the survey will take only several minutes. To make sure this survey is anonymous, you can deliver the survey back to me in an envelope. If you would like to know more about this survey, you may contact me via email ________________________ or telephone _________________.**

**DATE:_____________________**

1. **ARE YOU: FEMALE 🞏MALE🞏**
2. **HOW OLD ARE YOU:**

**🞏18 - 25 years**

**🞏26-45 years**

**🞏46-65 years**

**🞏66-80 years**

**🞏81-95 years**

**🞏96 years and older**

1. **ARE YOU**

**🞏STUDYING ABOUT A HEALTHCARE (I.E. MEDICINE, PHARMACY)**

**🞏STUDYING OUTSIDE A HEALTHCARE SETTING**

**🞏WORKING / WORKED IN A HEALTHCARE SETTING**

**🞏WORKING / WORKED OUTSIDE A HEALTHCARE SETTING**

**🞏UNEMPLOYED**

**🞏OTHER (PLEASE SPECIFIY)______________________________**

1. **a) AT WHAT WARD WERE YOU HOSPITALIZED (PLEASE SPECIFIY):**

**­­­­­­­­­­­­­­­­­___________________________________________________________________________**

1. **WHAT WAS THE NATURE OF YOUR ADMISSION TO HOSPITAL FOR THIS VISIT?**

**🞏SURGICAL**

**🞏NON-SURGICAL**

**🞏I DO NOT KNOW**

1. **a) WERE YOU TRANSFERED FROM ANOTHER HOSPITAL? 🞏YES 🞏NO**

**b) IF ANSWER IS YES, WHAT EAS THE REASON OF YOUR TRANSFER (I.E. CONTINUED MEDICAL CARE? ­­­­­­­­­­­­­­­­­­­­­­­­­­­________________________________________________________________________________**

1. **DO YOU KNOW WHAT A DRUG SHORTAGE IS?🞏YES 🞏NO If your answer is yes, please go to question 7. For the answer NO, go to the question 8.**
2. **BY YOUR OWN WORDS, PLEASE WRITE WHAT IS A DRUG SHORTAGE?**

**___________________________________________________________________________**

**FOR THE PURPOSE OF THIS STUDY A DRUG SHORTAGE IS insufficient supply (i.E. from hospital pharmacy) of a drug that you are taking without generic subsitute. Generic substitute is a drug with the same active substance as the drug you are taking but from another manufacturer.**

1. **IN YOUR OPINION, WERE YOU AFFECTED BY A DRUG SHORTAGE WHILE IN THE HOSPITAL?**

**🞏YES 🞏NO 🞏I DO NOT KNOW If your answer is yes, please go to question 9. For the rest ofanswers go to the question 13.**

1. **a) WERE YOU INFORMED BY A HEALTHCARE PROVIDER (PHYSICIAN, PHARMACIST, NURSE OTHER ) ABOUT DRUG SHORTAGE?**

**🞏YES 🞏NO 🞏I DO NOT KNOW**

**b) IF YOUR ANSWER IS YES, PLEASE SPECIFY BY WHO:**

**🞏 PHYSICIAN 🞏 PHARMACIST 🞏 NURSE 🞏OTHER (PLEASE SPECIFIY)_______________**

1. **a) DID A HEALTHCARE PROVIDER (PHYSICIAN, PHARMACIST, NURSE, OTHER) PROVIDED YOU WITH THE INFORMATION ABOUT POSSIBLE SUBSTITUTION FOR THE DRUG SHORTAGE?**

**🞏YES 🞏NO 🞏THERE WAS NO SUBSTITUTION FOR MY DRUG 🞏I DO NOT KNOW**

**b) IF YOUR ANSWER IS YES, PLEASE SPECIFY BY WHO:**

**🞏 PHYSICIAN🞏 PHARMACIST🞏 NURSE 🞏OTHER (PLEASE SPECIFIY)_______________**

**c)WERE YOU SATISFIED WITH THE INFORMATION ABOUT A DRUG SHORTAGE FROM THE HEALTHCARE PROVIDER (PHYSICIAN, PHARMACIST, NURSE, OTHER)?**

**🞏YES 🞏NO🞏I DO NOT KNOW🞏NO OPINION**

1. **a) IF THERE WAS NO SUBSTITUTION FOR YOUR DRUG, WAS YOUR TREATMENT:**

**🞏CANCELLED 🞏POSTPONED 🞏I DO NOT KNOW**

**b) IF YOUR TREATMENT WAS POSTPONED, FOR HOW MANY DAYS, WEEKS OR MONTHS?**

**___________________________________________________________________________**

1. **ARE YOU STILL EXPECTING TO GET A CALL WHEN YOUR TREATMENT WILL START AND HOW MANY DAYS, WEEKS OR MONTHS ARE YOU WAITING FOR THAT CALL?**

**___________________________________________________________________________**

1. **a) WHAT WAS A DURATION OF A DRUG SHORTAGE**

**🞏SHORT TERM (7 DAYS OR SHORTER)**

**🞏 BETWEEN 7 AND 14 DAYS**

**🞏 BETWEEN 15 AND 28 DAYS**

**🞏 BETWEEN 1 AND 3 MONTHS**

**🞏 LONGER THAN 3 MONTHS**

**🞏I DO NOT KNOW**

**b) DID IT AFFECT YOUR HEALTH (I.E. YOUR HEALTH WORSENED – YOUR SUGAR LEVEL WAS HIGH, YOU FELT DIZZY, YOU GOT A HEADACHE)?**

**🞏YES 🞏NO🞏I DO NOT KNOW**

1. **WHAT DO YOU THINK HOW DRUG SHORTAGE DID OR CAN AFFECT YOUR HEALTH?**

**___________________________________________________________________________**

**___________________________________________________________________________**

1. **IF THERE IS A DRUG SHORTAGE WITHOUT A GENERIC SUBSTITUTE* AVAILABLE, WOULD YOU LIKE TO BE INFORMED ABOUT ALTERNATIVE TREATMENT OPTION(S)?**

**🞏YES 🞏NO 🞏I DO NOT KNOW**

1. **WOULD YOU LIKE TO HAVE MORE INFORMATION ON DRUG SHORTAGES?**

**🞏YES 🞏NO 🞏I DO NOT KNOW**

1. **WOULD YOU PREFER INFORMATION ON DRUG SHORTAGES (tick all that apply):**

**🞏INFORMATION BY A HEALTHCARE PROVIDER (PHYSICIAN, PHARMACIST, NUSRE, OTHER ) PLEASE SPECIFY BY WHO: 🞏 PHYSICIAN 🞏 PHARMACIST 🞏 NURSE 🞏OTHER (PLEASE SPECIFIY)_______________**

**6🞏ON A NATIONAL TV COMMERCIAL**

**🞏LOCAL OR NATIONAL NEWSPAPERS**

**9🞏FROM THE WEBSITE OF THE NATIONAL AUTHORITY**

**🞏 FROM THE HOSPITAL WEBSITE**

**8🞏INFORMATION LEAFLET**

**4🞏ALL THE ABOVE**

**7🞏NONE OF THE ABOVE**

**3🞏I DO NOT KNOW**

**5🞏I DO NOT PREFER INFORMATION ABOUT DRUG SHORTAGES**

***Please read the following statement and check the box if you agree:***

***□  I, patient at __________________hospital, agree that these data may be processed anonymously for research purposes***

*** GENERIC SUBSTITUTE – it is a drug with the same active substance as the drug you are taking but from another manufacture**
