# Supplementary material for: Patients perspectives on drug shortages in six European hospital settings – a cross sectional study
Source: BMC Health Serv Res. 2021 Jul 12;21:689. doi: 10.1186/s12913-021-06721-9 (PMC8274960; doi:10.1186/s12913-021-06721-9)
Supplement: Supplementary file 2 — Additional file 2. [file 12913_2021_6721_MOESM2_ESM.docx]

**Patients Perspectives on Drug Shortages in six European hospital settings**

**Darija Kuruc Poje^1*^, Domagoj Kifer^2^, Isabelle Huys^3^, Joao Miranda^4,5^, Helena Jenzer^6^, Nenad Miljković^7^, Torsten Hoppe-Tichy^8^, Marcin Bochniarz^9^, Roberto Frontini^10^, David G Schwartz^11^, Vesna Vujić-Aleksić^12,13^, Lana Nežić^13^, Eleni Rinaki^14^, Leonidas Tzimis^14^, Kim Green^8^, Jelena Jovanić^15,16^, Bojana Carić^15, 17^, Danijela Mandić^15,18^, Katarina Vilić^1^, Tomasz Bochenek^19^, Vesna Bačić Vrca^20^, Srećko Marušić^21^**

1. Pharmacy Department, General hospital “dr. Tomislav Bardek”, Koprivnica, Croatia
2. Department of Biophysics, Faculty of Pharmacy and Biochemistry, University of Zagreb, Zagreb, Croatia
3. Department of Pharmaceutical and Pharmacological Sciences, KU Leuven, Leuven, Belgium
4. Departamento de Tecnologias, Escola Superior de Tecnologia e Gestão, Instituto Politécnico de Portalegre, Portalegre, Portugal
5. CERENA - Centro de Recursos Naturais e Ambiente, Instituto Superior Técnico, Universidade de Lisboa, Lisboa, Portugal
6. Berner Fachhochschule Health Professions Ernährung und Diätetik, Zürich, Switzerland
7. Pharmacy Department, Institute of Orthopaedic Surgery “Banjica”, Belgrade, Serbia
8. Pharmacy Department, Heidelberg University Hospital, Heidelberg, Germany
9. Specialist Hospital Brzozów, Podkarpackie Oncological Center, Brzozów, Poland
10. European Association of Hospital Pharmacists (EAHP), Brussels, Belgium
11. School of Business, Bar-Ilan University, Ramat Gan, Israel
12. Republic of Srpska Agency for Certification, Accreditation and Quality Improvement in Health Care, Banja Luka, Bosnia and Herzegovina,
13. Department of Pharmacology, Toxicology and Clinical Pharmacology, Faculty of Medicine, University of Banja Luka, Banja Luka, Bosnia and Herzegovina
14. Hospital pharmacy, Chania General Hospital “Saint George”, Chania, Crete, Greece
15. Faculty of Medicine, University of Banja Luka, Banja Luka, Bosnia and Herzegovina
16. Department of Cardiology, University Clinical Centre of Republic of Srpska, Banja Luka, Bosnia and Herzegovina
17. Department of Endocrinology, University Clinical Centre of Republic of Srpska, Banja Luka, Bosnia and Herzegovina
18. Department of Hematology, University Clinical Centre of Republic of Srpska, Banja Luka, Bosnia and Herzegovina
19. Department of Drug Management, Faculty of Health Sciences, Jagiellonian University Medical College, Kraków, Poland
20. Pharmacy Department, Clinical hospital Dubrava, Zagreb, Croatia
21. Endocrinology Department, Clinical hospital Dubrava, Zagreb, Croatia

*** Correspondence:**Darija Kuruc Poje, darija_kuruc@yahoo.com, ORCID ID: 0000-0002-7893-786X

# Supplementary material

| Table 2. Occupation: healthcare related or non-healthcare related | | |
| --- | --- | --- |
| Country specific hospitals | Healthcare related (eg, studying or working in medicine, pharmacy)  N (%) | Non - healthcare related (eg, studying or working outside healthcare setting, unemployed)  N (%) |
| Greece (H-GR) | 8 (5) | 108 (93) |
| Croatia (H-CR) | 3 (3) | 110 (97) |
| Bosnia and Herzegovina (H-BiH) | 15 (14) | 90 (86) |
| Poland (H-PO) | 11 (11) | 93 (89) |
| Serbia (H-SE) | 2 (2) | 98 (98) |
| Germany (H-GE) | 8 (12) | 61 (88) |
| Total N (%) | 47 (8) | 560 (92) |

Table 3. Hospital ward and transfer reasons

|  | Hospitalized ward | | | Transfer | | Reason for transfer | | | | |
| --- | --- | --- | --- | --- | --- | --- | --- | --- | --- | --- |
| Country specific hospitals | Surgical N (%) | Non-surgical N (%) | I do not know N (%) | Yes N (%) | No N (%) | Continued medical treatment N (%) | Additional diagnostics N (%) | Treatment not available /possible in other institutions N (%) | Emergency / closer to place of stay N (%) | No answer N (%) |
| Greece (H-GR) | 69 (59) | 47 (41) | 0 (0) | 2 (2) | 114 (98) | 1 (1) | 1 (1) | 0 (0) | 0 (0) | 0 (0) |
| Croatia (H-CR) | 71 (63) | 42 (37) | 0 (0) | 1 (1) | 112 (99) | 1 (1) | 0 (0) | 0 (0) | 0 (0) | 0 (0) |
| Bosnia and Herzegovina (H-BiH) | 20 (19) | 67 (64) | 18 (17) | 16 (15) | 89 (85) | 14 (13) | 2 (2) | 0 (0) | 0 (0) | 0 (0) |
| Poland (H-PO) | 24 (23) | 77 (74) | 3 (3) | 15 (14) | 89 (86) | 12 (12) | 2 (2) | 0 (0) | 0 (0) | 1 (1) |
| Serbia (H-SE) | 80 (80) | 20 (20) | 0 (0) | 4 (4) | 96 (96) | 4 (4) | 0 (0) | 0 (0) | 0 (0) | 0 (0) |
| Germany (H-GE) | 46 (67) | 21 (30) | 2 (3) | 15 (22) | 54 (78) | 1 (1) | 2 (3) | 6 (9) | 6 (9) | 0 (0) |
| Total N (%) | 310 (51) | 274 (45) | 23 (4) | 53 (9) | 554 (91) | 33 (5*) | 7 (1*) | 6 (1*) | 6 (1*) | 1 (0*) |

*Percentage from overall (N=607) number of patients
